# Outcomes of acetabular fractures treated with acute fix and replace versus open reduction and internal fixation in elderly population: a multicentric retrospective study

**DOI:** 10.1007/s00264-022-05535-6

**Published:** 2022-08-11

**Authors:** Amarildo Smakaj, Giuseppe Rovere, Dalila Scoscina, Domenico De Mauro, Rocco Erasmo, Concetto Battiato, Giulio Maccauro, Francesco Liuzza

**Affiliations:** 1 Department of Geriatrics and Orthopaedic Sciences, Università Cattolica del Sacro Cuore, Rome, Italy; 2 Department of Aging, Neurological, Orthopaedic and Head-Neck Sciences, Fondazione Policlinico Universitario Agostino Gemelli IRCCS, Rome, Italy; 3 Department of Clinical and Molecular Sciences, Università Politecnica Delle Marche, Torrette Di Ancona, Italy; 4 Orthopedics and Traumatology, Ospedale Santo Spirito, Pescara, Italy; 5 Orthopedics and Traumatology, ASUR Marche Area Vasta 5, Ospedale Mazzoni, Ascoli Piceno, Italy

**Keywords:** Acetabular fracture, Elderly patients, Combined hip procedure, CHP, Acute fix and replace

## Abstract

**Purpose:**

The optimal operative treatment for displaced acetabular fractures in elderly population is still object of debate. Acute fix and replace procedure, the so called “combined hip procedure” (CHP), was introduced because of the poor results of the open reduction and internal fixation (ORIF) alone. The aim of the study is to compare clinical outcomes of CHP and ORIF alone for the treatment of acetabular fractures in elderly patients.

**Methods:**

This is the largest multicentric retrospective analytical study, with a case–control design on the issue. Hospital records and clinical notes were reviewed to collect demographic, peri-operative, and clinical data.

**Results:**

A total of 45 patients met the inclusion criteria: 24 patients entered the CHP group whereas 21 entered the ORIF control group. The mean age was 69.5 +  − 1.12 years in the ORIF group and 73.4 +  − 1.84 in the control group. The most frequent traumatic mechanism was the fall from same level in both groups (37.5% CHP; 42.9% ORIF). Operating time was significantly lower in the CHP group compared to the ORIF group (207 +  − 11.0 ORIF; 175 +  − 9.16 CHP; *p* < 0.05). Moreover, full weight-bearing was allowed significantly earlier in the CHP group compared to ORIF alone (37.3 +  − 1.59 ORIF; 32.5 +  − 1.69 CHP; *p* < 0.05). Among the clinician-completed scores, the HHS at three months was higher in the CHP group (66.3 +  − 1.83 ORIF;73.6 +  − 2.09 CHP; *p* < 0.05). All the other clinical outcomes were similar in both study groups.

**Conclusion:**

CHP is desirable treatment option in elderly patients with acetabular fracture when there are poor expected outcomes in terms of joint survival with ORIF alone.

## Introduction

Epidemiological studies have reported an increase up to 30% in the incidence of acetabular fractures among elderly population in the past decades [1–4]. The most common mechanisms of injury in these patients are low-energy fragility traumas, frequently occurring as fall on the same level [4, 5]. However, the modern geriatric patients are not free from high-energy trauma mechanisms such us motor vehicular accidents which represents an overall 28.73% of injuries [5]. The increasing mean age of acetabular fractures has raised as a public health issue because they are associated with significant injury-induced morbidity and mortality rate [6]. The optimal operative treatment for displaced acetabular fractures in elderly patients remains still object of debate [7–9]. Open reduction and internal fixation (ORIF) may not be an effective alternative to achieve a satisfactory acetabular fracture repair, especially in elderly patients [10, 11]. In fact, it was reported a rate of conversion to arthroplasty after ORIF of 28% in medically stable geriatric patients [12]. Poor clinical results after ORIF led to introduce acute fix and replace procedure for the treatment of acetabular fractures in elderly population [13]. A recent systematic review reported advantages using ORIF together with simultaneous acute total hip arthroplasty (THA) in appropriately selected patients [14], a treatment strategy named “combined hip procedure” (CHP). To date, the existing literature concerning the outcomes in patients treated by CHP is limited, and studies are required to help surgeons in the appropriate treatment choice. As far as we know, we present the largest cohort study of elderly patients with acetabular fracture treated with CHP and analyzed in a comparative setting with patients treated with ORIF alone.

The purpose of this study is to evaluate if CHP provides better outcomes than ORIF alone for the treatment of displaced acetabular fractures in patients older than 60 years old.

## Patients and methods

### Patients and outcome measures

This is a multicentric retrospective analytical study, with a case–control study design. This study was performed in line with the principles of the Declaration of Helsinki. As this is an observational study, local Ethics Committees have confirmed that no ethical approval is required. All patients older than 60 years with acetabular fractures treated with CHP were identified from trauma databases and included in the study. Patients were excluded in case of incomplete clinical and radiological data, follow-up shorter than two years, previous fixation of proximal femur, previous hemiarthroplasty, or THA. CHP was performed in case of displaced and comminuted acetabular fracture with poor expected prognosis because of significant acetabular impaction, associated femoral head fractures, and/or preexisting severe osteoarthritis. Patients with similar acetabular fracture patterns, without relevant osteoarthritis, entered the ORIF control group in order to compare clinical outcomes.

Hospital records and clinical notes were reviewed to collect demographic data, injury mechanism, date of surgery, operating times, peri-operative bleeding, peri-operative and post-operative complications, type of implants, weight-bearing timing, and thromboprophylaxis.

Acetabular fractures were classified according to Judet and Letournel [15] by two senior orthopaedic surgeons (C.B., F.L.) separately: there was 100% classification concordance.

Clinical and radiological follow-ups were collected at three months, six months, one year, and then every year after surgery. Clinical outcomes were evaluated with patient reported outcomes (PROMs) and clinical completed scores. Quality of life (QoL) was assessed using 12-item short-form health survey questionnaire (SF-12) and pelvic discomfort index (PDI) [16]. Clinician-completed scores included Harris Hip Score (HHS) and Modified Merle d’Aubigné and Postel Method [17].

All patients were evaluated with pre-operative and follow-up anteroposterior, obturator, and iliac oblique radiographs. Computed tomography and three-dimensional computed tomography reconstructions were performed for pre-operative surgical planning.

Routine follow-up radiographs were assessed for consolidation or non-union, implant loosening according to Engh score, and presence of heterotopic ossifications in accordance with Brooker classification [18]. Secondary osteoarthritis in the ORIF group was evaluated according to Kellgren and Lawrence [17].

### Surgical technique and implants

Surgical approaches were selected case by case depending on type of fractures in order to give proper exposure of the surgical site. In particular, the Kocher-Langenbeck approach (in lateral decubitus) was always performed for CHP to complete THA. This surgical approach was ideal when the posterior column and wall of the acetabulum were fractured. Otherwise, modified Stoppa and ilioinguinal approaches were performed in case of involvement of the anterior acetabular structures implanting suprapectineal quadrilateral surface plates in case of comminuted fragments of the quadrilateral lamina [19]. Operations were conducted by three senior orthopaedic consultants, experienced in pelvic surgery (C.B., F.L., R.E.). In the CHP group, 14 patients were operated with a unique Kocher-Langenbeck approach whereas a total of seven patients underwent modified Stoppa or ilioinguinal approach too. In the ORIF group, eight fractures were treated with Kocher-Langenbeck approach and seven fractures with modified Stoppa plus the later window of the ilioinguinal approach. In nine cases, combined approaches were mandatory to obtain a good reduction and osteosynthesis.

**Fig. 1:**
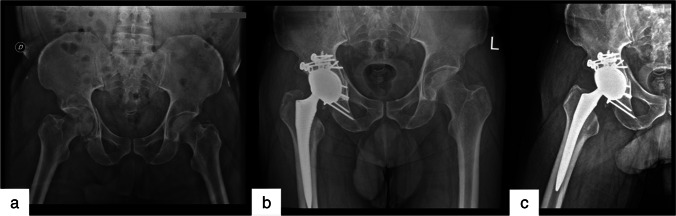
Pelvic x-rays of a 64-year-old man with transverse + posterior wall fracture of the right acetabulum (**a**). CT scan revealed negative prognostic factors (posterior wall comminution, marginal impaction, femoral head impactions). CHP was performed restoring the overall shape of the acetabulum with double plating and implanting a multihole revision acetabular component (**b**, **c**)

Matta Pelvic System (MPS) was used to perform ORIF in both groups. When osteosynthesis and acetabular bone stock were adequate, primary implants were used in the CHP group. The Burch-Schneider rig was implanted only in three acetabular fractures. An explicative case of CHP is shown in Fig. 1a–c.

### Statistical analysis

Data were analyzed for descriptive statistics as mean or median for continuous variables and frequency distribution (%) for categorical variables. Student’s *t* test was used to investigate differences between CHP and control groups for continuous data. Fisher’s exact test and the chi-square test were used to study categorical variables.

## Results

Since 2018 until the spread of SARS-Cov2 pandemic, 61 consecutive acetabular fractures in patients older than 60 years were identified in the included centers. Among these patients, nine cases were excluded from the study because of incomplete clinical and radiological data and seven were lost in the follow-up. A total of 45 patients met our inclusion criteria and were enrolled in the current study. 24 patients entered the CHP group whereas 21 entered the ORIF control group. The mean age was 69.5 +-1.12 years in the ORIF group and 73.4 +- 1.84 in the control group, but there were not statistically differences between the two study groups (p=0.07). Demographic characteristics of patients are reported in Table 1.

**Table 1:** Demographic, trauma mechanism and surgical treatment data.

	ORIF	CHP	P value
Number of Patients	24	21	
Male/female Ratio	1:2	5:16	
Age	69.5 +-1.12	73.4 +- 1.84	0.07
BMI	24.4 +- 1.05	25.2 +- 1.03	0.50
Injury Mechanism			
Traffic Accident	16.7% (n=4)	14.3% (n=3)	
Pedestrian Hit by Car	25.0% (n=6)	23.8% (n=5)	
Fall From High	12.5% (n=3)	14.3% (n=3)	
Fall From Same Level	37.5% (n=9)	42.9% (n=9)	
Others	8.3% (n=2)	4.8% (n=1)	
Associated lesions			
Hip dislocation	5	9	
Concomitant fractures	12	11	
Head injuries	6	3	
Thoracic injuries	3	-	
Abdominal injuries	1	-	
Vascular injuries	2	1	
Others	4	3	
ASA Classification*			
I	2	1	
II	17	19	
III	5	1	
Time to Operation (days)	6.25 +- 3.17	8.05 +- 3.85	0.09
Operating Time (minutes)	207 +- 11.0	175 +- 9.16	0.03†
Blood Loss (mL)	624 +- 31.0	588 +- 31.1	0.41
Blood Transfusion (n)	75,0% (n= 18)	85.7% (n=18)	0.46
Time of Hospitalization (days)	16.0 +- 1.02	14.8 +- 1.53	0.50
Full Weight Bearing (days)	37.3 +- 1.59	32.5 +- 1.69	0.04†

*American Society of Anesthesiologists classification

†Statistically significant, p< 0.05

The most frequent traumatic mechanism was the fall from same level in both groups (37.5% CHP; 42.9% ORIF) followed by pedestrian hit by car (23.8% CHP; 25.0% ORIF).

As concerns associated lesions, concomitant fractures have the higher incidence among the enrolled patients (12 cases in the CHP group; 11 in the ORIF group). Further associated injuries are summarized in Table 1. The general health conditions of the included patients were similar in both groups as there are not significant differences in the ASA (American Society of Anesthesiologists) classification.

Acetabular fractures classification of the enrolled patients is shown in Table 2. Anterior column with posterior hemi transverse is the most frequent type of fracture in both groups, followed by posterior wall and column.

**Table 2:** Acetabular fractures classification (according to Judet-Letournel)

	ORIF	CHP
Posterior wall	1	2
Posterior column	2	1
Anterior wall	1	0
Anterior column	1	1
Transverse	2	2
Posterior wall and column	3	3
Transvers and posterior wall	1	1
T-shaped	2	2
Anterior column with posterior hemi transverse	8	7
Both columns	3	2
Tot	24	21

The majority of the intra and peri-operative findings were similar in both groups. In fact, time to operation, blood loss, blood transfusion, and time of hospitalization showed no statistically significant differences (*p* > 0.05) (Table 1). On the contrary, operating time was significantly lower in the CHP group compared to the ORIF group (207 +  − 11.0 ORIF; 175 +  − 9.16 CHP; *p* < 0.05). Moreover, full weight-bearing was allowed statistically earlier in the CHP group compared to ORIF alone (37.3 +  − 1.59 ORIF; 32.5 +  − 1.69 CHP; *p* < 0.05). Complications are listed in Table 3. No re-operations were required in the CHP group whereas seven patients among the ORIF group developed secondary osteoarthritis, demanding for total hip arthroplasty (THA). No patients died within the follow-up period. There were no deep infections or non-unions detected. Secondary osteoarthritis occurred in the 29.2% of the ORIF group. There were no intra-operative complications recorded. The physical and mental component scores of the SF-12 improved during the follow-up period in both groups (Table 4) but no significant difference was detected (*p* > 0.05). The patient-reported pelvic discomfort index (PDI) was recorded only two years after surgery as it was not routinely performed during follow-up. This PROMs showed better outcomes in the CHP group, but the difference was not significant (*p* > 0.05). Among the clinician-completed scores, the HHS at three months was higher in the CHP group (66.3 +  − 1.83 ORIF; 73.6 +  − 2.09 CHP; *p* < 0.05). The MAPM was higher in the CHP group but no significant difference emerged.

**Table 3:** Complications

	ORIF	CHP	
Implant loosening*	4.2% (*n* = 1)	9.5% (*n* = 2)	*p* > 0.05
Non-union	-	-	
Heterotopic ossification**	20.8% (*n* = 5)	14.3% (*n* = 3)	*p* > 0.05
Secondary osteoarthritis	29.2% (*n* = 7)	/	*p* > 0.05
Wound infection	12.5 (*n* = 3)	14.3% (*n* = 3)	*p* > 0.05
Deep infection	-	-	
DVT	8.3% (*n* = 2)	14.3% (*n* = 3)	*p* > 0.05
Others	8.3% (*n* = 2)	4.8% (*n* = 1)	*p* > 0.05

^*^Engh classification

^**^Brooker classification

**Table 4:** Clinical outcomes

		3 m			6 m			2 y		
		ORIF	CHP	*p* value	ORIF	CHP	*p* value	ORIF	CHP	*p* value
SF-12	PCS**	35.8 + − 0.67	36.5 + − 0.79	0.49	39.9 + − 0.58	41.3 + − 0.61	0.10	40.6 + − 0.54	41.3 + − 0.86	0.51
	MCS^‡^	33.5 + − 0.65	32.3 + − 0.63	0.20	38.6 + − 0.54	39.5 + − 0.56	0.27	40.4 + − 0.42	41.5 + − 0.41	0.07
Pelvic Discomfort Index (PDI)								53.4 + − 1.82	57.9 + − 1.34	0.06
Harris Hip Score (HSS)		66.3 + − 1.83	73.6 + − 2.09	0.01†	77.3 + − 2.72	82.7 + − 2.36	0.15	78.9 + − 2.40	83.8 + − 2.42	0.22
MAPM*		8.21 + − 0.27	9.00 + − 0.34	0.07	8.54 + − 0.31	9.05 + − 0.37	0.28	8.83 + − 0.38	8.95 + − 0.36	0.83

^*^Modified Merle d’Aubigné and Postel score

^†^Statistically significant *p* < 0.05

^**^Physical component score

^‡^Mental component score

## Discussion

Our results demonstrated good clinical outcomes in both study groups after acetabular fracture in elderly patients. The cohort study can be considered similar in both groups as they do not significantly differ in terms of demographics such as mean age, M/F ratio, ASA classification, and BMI. However, it is not possible to exclude any selection bias. In fact, the allocation of patients in the ORIF or acute fix and replace group was driven by the operating surgeon’s expectation on the joint survival. In particular, surgeons followed the hospital for special surgery treatment algorithm for acetabular fractures in elderly patients [20], identifying the presence of negative prognostic factors for the development of secondary osteoarthritis. The main elements taken into consideration were marginal impaction, posterior wall comminution, roof arch angle < 45°, hip dislocation, preexisting arthrosis, and femoral head fracture or impaction [20–23]. The operating time was significantly lower in the CHP group which reduces the patient’s surgical distress, and it may represent an advantage in terms of cost efficiency. We suppose that patients allocated in the ORIF group required longer operating time because the procedure needs anatomical reduction of the acetabular surface to be effective and this is often challenging to be achieved. Moreover, our results revealed that weight-bearing was allowed significantly earlier in the CHP group, certainly reducing bed rest complications [24, 25].

**Fig. 2:**
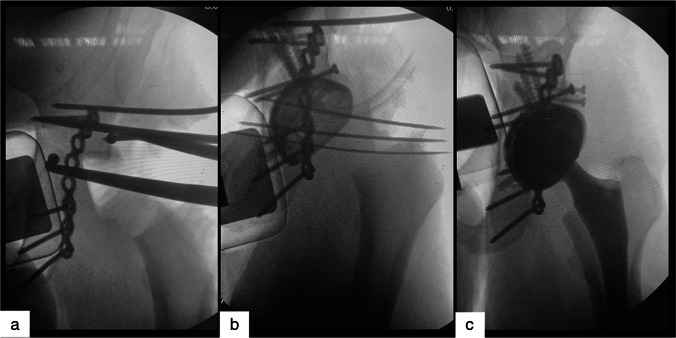
Proposed surgical steps for CHP: appropriate osteosynthesis restoring as much as possible the pre-injury acetabular shape and dimension (**a**), reaming and multihole revision acetabular component impaction with screws to achieve a third acetabular stabilization anchor (**b**), and correct orientation of the acetabular and femoral components (**c**)

Early weight-bearing is possible if total hip replacement is preceded by a stable osteosynthesis. Manson suggested that the critical bony buttresses for acetabular component stability are the subchondral bone attached to the anterior inferior iliac spine (AIIS) and the subchondral bone attached to the posterior column [26]. However, the relationship between these two parts of the acetabulum is often disrupted by the fracture. Therefore, the aim of the osteosynthesis in CHP is to restore the relationship between the AIIS and the ischium before reaming in order to achieve the pre-injury acetabular dimensions (Fig. 2a). It would be ideal to impact multihole revision acetabular components which allows to use screws to achieve a third acetabular stabilization anchor (Fig. 2b, c). Similarly, the optimum construct for acute fix and replace procedure was already debated by Rickmann M et al. [27] who suggested creating an A-construct: the concept is that of creating an A-frame equivalent, by stabilizing both anterior and posterior columns, followed by using the acetabular shell as the cross-piece. When properly performed, CHP allows earlier weight-bearing and satisfactory clinical outcomes. Moreover, using available arthroplasty registry data, recent review of the literature estimated that about three-quarters of hip replacements last 15–20 years and just over half of hip replacements last 25 years [28]. Therefore, we can assume that the CHP will probably be the only surgery elderly patients will undergo because of their acetabular fracture. Several retrospective studies investigated the feasibility and safety of the acute fix and replace procedure [29–32], but there are few available case–control studies [33, 34]. Borg et al. [33] already investigated the re-operation rates after CHP in a comparative setting with ORIF procedure alone, demonstrating that the need for secondary surgery was markedly reduced without an obvious increase in peri-operative mortality. However, the main limitation of this study is the low sample size: 13 patients allocated in the CHP group and 14 in the ORIF with 5 dropouts at the last follow-up. In 2015, a systematic review by Jauregui JJ et al. [14] assessed the reported demographics, fracture patterns, surgical approaches, fixation methods, outcomes, and complications of the CHP, but there was not any comparison between surgical treatment option.

In our experience, apart from the 3 months of clinical follow-up which revealed higher HHS in patients treated with CHP (Table 4), all the other clinical outcomes were similar in both study groups. However, patients treated with acute fix and replace showed better clinical scores, even if there was not any statistically significant difference. We cannot exclude type β statistical errors which means that there are differences between the two groups which were not detected due to the power of the study. An adequately powered randomized controlled trial, such as the AceFIT study (ISRCTN16739011), is required to determine which is the optimal treatment in elderly population with acetabular fracture. Preliminary results of a feasibility study have been presented at the Orthopaedic Trauma Association (OTA) 2022 annual meeting by Carrothers AD et al. [35] but results are expected in the next years. Geriatric Acetabular fracTures (GATOR) [36] is a also a promising observational cohort study involving academic institutions affiliated with the Canadian Orthopaedic Trauma Society (COTS).

The main limitation of the present study is its retrospective nature which enables any kind of randomization with possible selection bias. Surgical procedures were not performed by a single surgeon and different types of prosthetic implants were used. Further subgroup analysis should be useful to define the ideal prosthetic implant in case of acetabular fracture, but larger cohort study is required.

## Conclusion

In the light of our results, which are consistent with the current literature, the CHP should be a desirable treatment option in elderly patients with acetabular fracture when there are poor expected outcomes in terms of joint survival. Larger randomized clinical trials are in progress, and they will hopefully strengthen current knowledges on the issue. However, further investigations are needed to reveal possible beneficial cost efficiency aspects of the CHP.

## Data Availability

Data are available under reasonable request to the corresponding author.
